# Association between Serum Uric Acid Levels and Bone Mineral Density in Postmenopausal Women: A Cross-Sectional and Longitudinal Study

**DOI:** 10.3390/healthcare9121681

**Published:** 2021-12-04

**Authors:** Soyeon Kang, Dongjin Kwon, Jiwoo Lee, Youn-Jee Chung, Mee-Ran Kim, Jeong Namkung, In Cheul Jeung

**Affiliations:** 1 St. Vincent’s Hospital, Department of Obstetrics and Gynecology, College of Medicine, The Catholic University of Korea, Seoul 06591, Korea; ksy@catholic.ac.kr (S.K.); cumckwon@catholic.ac.kr (D.K.); jwlee_90@naver.com (J.L.); 2 Seoul St. Mary’s Hospital, Department of Obstetrics and Gynecology, College of Medicine, The Catholic University of Korea, Seoul 06591, Korea; porshe80@catholic.ac.kr (Y.-J.C.); mrkim@catholic.ac.kr (M.-R.K.); 3 Eunpyeong St. Mary’s Hospital, Department of Obstetrics and Gynecology, College of Medicine, The Catholic University of Korea, Seoul 06591, Korea; rossa@catholic.ac.kr; 4 Daejeon St. Mary’s Hospital, Department of Obstetrics and Gynecology, College of Medicine, The Catholic University of Korea, Seoul 06591, Korea

**Keywords:** menopause, antioxidant, uric acid, bone mineral density, hormone replacement therapy

## Abstract

Background: Uric acid is one of natural antioxidants in human body. There have been several studies on the correlation between uric acid with oxidative stress and osteoporosis. However, the data are insufficient and results are controversial. In this regard, we determined the association between uric acid levels and bone mineral density (BMD) during the postmenopausal period. Methods: We analyzed data from 328 postmenopausal women (mean age, 57.3 ± 6.5 years; mean serum uric acid level, 4.6 ± 1.0 mg/dL). The participants were divided into three groups based on tertiles of the serum uric acid level. The participants receiving hormone replacement therapy (HRT), bisphosphonates, or lipid-lowering agents were included. Results: Blood urea nitrogen, serum creatinine, and serum triglyceride levels were significantly higher in the upper tertiles of uric acid levels. No significant difference was found in the mean uric acid levels between medication users and non-users. Each HRT regimen had a different mean serum uric acid level. A cross-sectional analysis showed no significant correlation between the serum uric acid levels and BMD in the spine and femoral neck (spine BMD: 1.050 ± 0.131, 1.060 ± 0.160, 1.084 ± 0.140, *p* = 0.22; femoral neck BMD: 0.837 ± 0.110, 0.849 ± 0.096, 0.863 ± 0.115, *p* = 0.28 for each tertile of uric acid). Longitudinal analysis of data from 186 women with follow-up examinations at a mean interval of 14.6 months also revealed no difference in reduction in both spine and femoral neck BMD between tertile groups of serum uric acid (the median BMD reduction for spine: −0.02, 0.01, −0.04, *p* = 0.95; the median BMD reduction for femoral neck: 0.008, 0.005, −0.003, *p* = 0.34). Conclusions: Serum uric acid level is not associated with BMD in postmenopausal women.

## 1. Introduction

Osteoporosis is a skeletal disease characterized by an increased risk of bone fracture resulting from an abnormality in bone strength. This disease is common in postmenopausal women. The risk factors for osteoporosis progression include aging, decreased physical activity, caffeine and alcohol intake, smoking, steroid use, thyroid disease, diabetes, and lack of estrogen, especially among menopausal women [1]. Estrogen deficiency is associated with oxidative stress; an experimental animal model study showed that antioxidants could prevent bone loss [2]. Natural antioxidants, including uric acid, bilirubin, and albumin, are present in humans. Studies have reported that uric acid removes free radicals, bilirubin suppresses oxidation, and albumin maintains the total antioxidant status [3,4,5]. Among these natural antioxidants, uric acid is the most abundant; it removes two-thirds of the free radicals from the plasma [3,6]. Several studies have investigated the association of uric acid with oxidative stress and osteoporosis. Uric acid levels of postmenopausal women were lower than those of perimenopausal women, and this difference affected the development of osteoporosis [7]. Moreover, the antioxidant effect of uric acid was more potent than that of vitamins or other enzymatic antioxidants [8].

Recently, studies have investigated the correlation between uric acid level and bone mineral density (BMD) in menopausal women. In a study of perimenopausal and postmenopausal women, BMD in several sites, fat mass, and lean mass of the entire body were higher in those with higher serum uric acid levels; and there were lower annual decreases in BMD of the lumbar spine and increased annual changes in lean body mass in those with higher serum uric acid levels [9]. Another study reported that high serum uric acid levels were correlated with a low risk of osteopenia and osteoporosis in postmenopausal women [10]. However, these results are not consistent with those of other reports. An observational analysis on menopausal women showed a strong correlation between bone-related outcomes and uric acid levels; however, the Mendelian randomization analysis did not reveal any causal relationship between these factors [11]. Furthermore, no significant correlation was found between uric acid level and BMD among women aged ≥30 years in a study of the National Health and Nutrition Examination Survey cohort in the United States from 2005 to 2010 after adjusting for confounding factors [12]. In an animal experiment of the same study, a comparison of BMD between hyperuricemic and normouricemic rats yielded a similar result, reporting no significant differences [12].

Furthermore, reports on the effect of hormone replacement therapy (HRT) or bisphosphonate use on serum uric acid levels are lacking despite the fact that both of these medications are frequently used for bone protection or osteoporosis treatment in menopausal women.

Therefore, we aimed to investigate the correlation between serum uric acid levels and BMD in postmenopausal women and the effect of HRT and bisphosphonate use on serum uric acid level.

## 2. Materials and Methods

### 2.1. Study Population and Baseline Characteristics

Healthy postmenopausal women who visited the outpatient gynecologic endocrinology clinic of St. Vincent’s Hospital for a regular gynecological check-up between 1 April 2013 and 31 March 2016 were considered for inclusion. Participants with diseases such as cancer, rheumatoid arthritis, and thyroid disease or those with prolonged steroid use were excluded as these conditions can affect bone metabolism. Smoking and alcohol intake could not be determined as relevant data were unavailable in their medical records. Altogether, 336 candidates were considered for the study. Among them, eight women without blood or BMD testing data were excluded.

We conducted a cross-sectional study of 328 healthy postmenopausal women whose last menstrual period was at least 1 year prior to their baseline annual gynecological check-up, with menopause confirmed by a serum concentration of the follicle-stimulating hormone (FSH) > 40 IU/L. They were divided into three groups according to the following tertiles of the serum uric acid level for easy comparison with the Australian study that conducted both longitudinal and cross-sectional analyses [9]: tertile 1, <4.1; tertile 2, ≥4.1 and <4.8; and tertile 3, ≥4.8 mg/dL. The Catholic Medical Center’s institutional review board waived the requirement for obtaining participants’ informed consent because of the study’s retrospective design. All research procedures were performed in accordance with relevant guidelines and regulations (IRB code: VC18RESI0258).

### 2.2. BMD Measurements

BMD of the lumbar spine from the L1 to the L4 level and of the femoral neck was measured using dual-energy X-ray absorptiometry and expressed as grams of bone mineral per square centimeter (g/cm^2^). Body mass index (BMI), body fat mass, abdominal obesity, and total lean body mass were measured with an InBody720 Body Composition Analyzer using the direct segmental multifrequency bioelectrical impedance analysis method (InBody Co., Ltd., Seoul, Korea).

### 2.3. Blood Sample Analysis

Serum glucose, urea nitrogen, creatinine, alkaline phosphatase, total cholesterol, triglyceride, low-density lipoprotein cholesterol, insulin, pyridinoline, osteocalcin, 25-hydroxyvitamin D, triiodothyronine, free thyroxine, thyroid-stimulating hormone, FSH, and estradiol levels were measured after the subjects fasted for at least 8 h.

### 2.4. Statistical Analyses

The data are presented as the means ± standard deviations (SDs) or the medians (ranges) for continuous variables and as numbers (percentages) for categorical variables. To confirm that continuous variables were normally distributed, the Kolmogorov–Smirnov test was applied. For the data that did not follow a normal distribution, the significance of the differences between groups was tested using the Kruskal–Wallis test with the Dwass–Steel–Critchlow–Fligner post hoc test. Simple and multiple linear regression analyses were performed, and the beta coefficients were determined to identify the degree of association between serum uric acid levels and BMD. Nonnormally distributed values were log-transformed before entering the model. Two-tailed *p*-values < 0.05 were considered statistically significant. The power was calculated using multiple linear regression analysis to establish a prediction equation for BMD with six predictors with an effect size f2 of 0.02 (small), alpha of 0.05, and sample size of 328, detecting a power of 88% (G*Power 3.1.9.7, Kiel University, Kiel, Germany). Statistical analyses were performed with SAS software version 9.4 (SAS Institute, Cary, NC, USA).

## 3. Results

The participants’ clinical characteristics are presented in Table 1. The participants’ mean age was 57.3 ± 6.5 years (range, 36–81 years), and the mean serum uric acid level was 4.6 ± 1.0 mg/dL (range, 2.2–8.0 mg/dL). There was no significant difference in the mean age among the three tertiles (*p* = 0.093). BMI was significantly higher in tertile 3 than in tertiles 1 and 2 (*p* = 0.001). Serum urea nitrogen, creatinine, and triglyceride levels were significantly higher in the upper tertiles (*p* = 0.013, *p* = 0.003, and *p* < 0.001 for tertiles 1, 2, and 3, respectively). The mean abdominal obesity was higher in tertile 3 than in the other tertiles (*p* = 0.055). The mean total lean body mass was higher in the upper tertiles than in the lower tertile (*p* = 0.148). The participants comprised 87 HRT (26.5%), 57 bisphosphonate (17.4%), and 84 lipid-lowering agents users (25.6%). No significant differences were found in the proportions of HRT, bisphosphonate, or lipid-lowering agents users between the tertiles (Table 1). No significant differences were found in the bone-related serum marker levels, including osteocalcin and 25-hydroxyvitamin D, between the tertiles. There were also no significant differences in FSH and estradiol levels between the tertiles (Table 1).

Table 2 displays the BMD distribution according to the uric acid levels at baseline and follow-up. At baseline, spine and femoral neck BMD were higher in the group with higher serum uric acid levels. The mean follow-up duration was 14.6 ± 2.9 months. The median changes in spine and femoral neck BMD, expressed as change from the baseline to the follow-up visit, were −0.001 and 0.005, respectively. In the spine, the median changes in BMD according to the serum uric acid level tertiles were −0.002 (tertile 1), 0.001 (tertile 2), and −0.004 (tertile 3) (*p* = 0.950). In the femoral neck, the median changes were 0.008, 0.005, and −0.003 (*p* = 0.343). There was a nonsignificant trend in the tertile 3 group towards a lower BMD loss in the femoral neck.

Data of serum uric acid, body fat mass, abdominal fatness, total lean body mass, and BMD were log-transformed for regression analysis because these data showed skewed distribution (Table 3). Age was negatively correlated and BMI was positively correlated with log-transformed lumbar spine BMD (β-coefficients = −0.003 ± 0.001, *p* = 0.027; β-coefficients = 0.02 ± 0.01, *p* = 0.003, respectively). Abdominal fatness had a strong negative correlation with lumbar spine BMD (β-coefficient = −0.72 ± 0.26, *p* = 0.005) (Table 3). In multiple linear regression analysis controlled for age, BMI, body fat mass, abdominal fatness, and total lean body mass, the adjusted β-coefficients of serum uric acid level with spine and femoral neck BMD were 0.01 ± 0.03 (*p* = 0.706) and −0.04 ± 0.04 (*p* = 0.327), respectively (Table 3).

There was no significant difference in the mean uric acid levels between users and non-users of HRT (*p* = 0.38), bisphosphonates (*p* > 0.99), or lipid-lowering agents (*p* = 0.57) (Table 4). Three regimens of HRT and one regimen of concurrent use of HRT with bisphosphonates were included. Twenty-eight participants received estrogen treatment (ET), 30 received estrogen and progesterone treatment (EPT), 13 received EPT with bisphosphonates, and 16 received tibolone. The mean serum uric acid level was 4.6 mg/dL among the 87 HRT users. The mean serum uric acid level for each HRT regimen was 4.9, 4.4, 4.6, and 4.7 mg/dL for ET, EPT, EPT with bisphosphonates, and tibolone, respectively (*p* = 0.28) (Table 4).

## 4. Discussion

We found no significant association between baseline serum uric acid levels and BMD, nor trend toward BMD changes according to serum uric acid levels in both spine and femoral neck. HRT users and non-users, as well as bisphosphonate users and non-users, did not feature a difference in the mean serum uric acid level. Each HRT regimen featured a different mean serum uric acid level and bisphosphonate users who concurrently used HRT had the mean serum uric acid level different from that of HRT users, though both of those findings were not significant. 

Uric acid, previously considered as a waste byproduct, is the final degradation product of purine metabolism. Hypouricemia is associated with multiple sclerosis [13]. Hyperuricemia is a condition in which the level of serum uric acid concentration is ≥6 mg/dL in women or ≥7 mg/dL in men in general [14], and it is associated with gouty arthritis, renal stones, cardiovascular disease, type 2 diabetes, and metabolic syndrome [15,16,17,18,19,20]. Hyperuricemia has different prevalence depending on the ethnicity, sex, region, and research design [21]; it is reported to be 11.4% in Korea [21] and up to 17% in some populations [22]. Overproduction of urate, which can be caused by tumor lysis syndrome and other diseases with high cellular turnover or genetic errors, is known to account for <10% of hyperuricemia cases [22]. Inefficient excretion of urate due to renal insufficiency or medications that impair renal urate clearance is known to account for about 90% of hyperuricemia cases in the general population [22]. Aging, weight gain, and excessive dietary consumption of alcohol or purine-rich foods, including red meat and seafood, may also increase serum uric acid level [22]. 

Uric acid has a proinflammatory function in the crystalline state. Its role in the soluble state has been debated; it was previously considered to be physiologically inactive, but is now recognized to have selective antioxidative effects in the serum at high physiological values (2.5–6.7 mg/dL), accounting for nearly half of all serum antioxidants in the human body [23,24,25,26]. Oxidative stress and low antioxidant levels are suggested to be correlated with low BMD and osteoporosis on the basis of the findings of several observational and epidemiologic studies [27,28,29]. In a cross-sectional study of men aged ≥70 years, high serum uric acid levels were correlated with high BMD at several skeletal sites, and there was low incidence of non-skeletal fractures after adjustments for multiple factors [30]. An observational study of perimenopausal and postmenopausal women showed comparable results [31].

Studies regarding uric acid levels and BMD in Korea have provided insufficient data and yielded conflicting results. In a retrospective study from a university hospital enrolling 2232 healthy women who underwent regular check-ups from 2010 to 2014, the correlation between uric acid levels and BMD was verified for both lumbar spine and femur of premenopausal women; however, it was not verified in postmenopausal women after adjustment for age and BMI [32]. Another study including 7502 healthy postmenopausal women who underwent regular check-ups from 2008 to 2010 reported that uric acid levels were positively correlated with all BMD measurements after adjustment for various factors [31].

A previous report suggested that hyperuricemia is associated with high BMD, possibly because the potential antioxidant effect of uric acid leads to the suppression of osteoclastic bone resorption; nevertheless, the antioxidant role of uric acid remains controversial [33]. Antioxidants may act as prooxidants in certain circumstances [34,35]. A previous review article investigating uric acid as a risk marker for cardiovascular disease, metabolic syndrome, and type 2 diabetes suggested that serum uric acid shows functional changes from antioxidant to prooxidant when its levels are elevated; moreover, uric acid levels are correlated with the progression of atherosclerosis from early to late stages [36]. In a recent article regarding uric acid and BMD in postmenopausal women with type 2 diabetes, uric acid levels were neither a protective factor nor a risk factor for osteoporosis of postmenopausal women with type 2 diabetes [37]. Uric acid may also act differently on BMD depending on ethnicity and race. A positive correlation between serum uric acid and lumbar BMD was suggested in another recent article among older individuals aged ≥60 years; however, this positive relationship was not found in Black people [38]. African Americans have more hyperuricemia and gout cases than European Americans, which is probably attributed to their higher rates of hypertension, obesity, and end-stage renal disease [39]. Serum uric acid could affect the bone differently according to individual circumstances, and there are many other bone-related parameters and unknown factors with a potential effect on a patient’s health status. It is difficult to identify the independent factors influencing BMD and determine their mechanisms of action because bone health is correlated with cardiovascular and metabolic status.

The mean age and serum uric acid level (57.3 ± 6.5 years and 4.6 ± 1.0 mg/dL, respectively) in our study were comparable with those in other studies [31,32,40]. However, the trend toward a positive correlation between serum uric acid levels and BMD observed in the cross-sectional analysis of other reports was not verified in this study. The duration of menopause, which is associated with BMD, averaged 8.9 years in our study, whereas this duration was not mentioned [9,10,11,31,32,41] or partially missing [40] in other studies. This study included 30 and 298 women who had experienced surgical and natural menopause, respectively. Four women with premature ovarian insufficiency experienced menopause before the age of 40 years, and 10 women experienced menopause before the age of 45 years. Most other studies did not clarify whether the study population included women with surgical menopause, early menopause, or premature ovarian insufficiency. The duration of menopause and proportion of women who experienced surgical or early menopause or premature ovarian insufficiency could contribute to the difference between our results and those of other studies.

One of the discriminative features of our study is that HRT and bisphosphonate users were included. The mean serum uric acid level was the highest in the ET group and the lowest in the EPT group. The mean serum uric acid level of the EPT plus bisphosphonate group was higher than that of the EPT group, but lower than that of the tibolone group. Our study was the first to investigate the relationship between postmenopausal bisphosphonate treatment and serum uric acid, showing results similar to those of a recent study on serum uric acid levels by postmenopausal HRT type [42]. Further studies are needed to explore the effects of bisphosphonates on serum uric acid levels in menopausal women. Few studies have reported the effects of HRT use on serum uric acid levels. A recent study on serum uric acid levels by postmenopausal HRT type reported that the lower serum uric acid levels in the EPT group compared with those in HRT non-users, ET users, and tibolone users at follow-up were due to the effects of progesterone, not estrogen [42]. Prior to this study, the effect of HRT on gout incidence in women aged ≥45 years was investigated, and it was found that there was a decreased odds ratio for gout in participants treated with EPT and tibolone; however, there was no change in the group treated with estrogen alone. The reduced odds ratio for gout may have been due to progesterone rather than due to estrogen [43]. In our study, 13 bisphosphonate users concurrently used EPT. The mean serum uric acid level in EPT plus bisphosphonate users was 4.6 mg/dL, which was lower than that in the ET-only users but higher than that in the EPT and HRT non-users. Although there are no comparable data from other studies, a previous study on the effects of two bisphosphonates on uric acid levels in participants with Paget’s disease reported that the serum uric acid levels were significantly lower after 6 months of bisphosphonate treatment in the initially hyperuricemic participants and returned to the initial values at 3 months after treatment cessation; the levels were unchanged by bisphosphonate treatment in the initially normouricemic participants [44]. The results of these two studies could be a signpost for future studies to identify the association between bisphosphonates and serum uric acid levels. 

Factors such as BMI, abdominal obesity, and triglycerides were positively correlated with serum uric acid levels. Abdominal obesity was negatively correlated with BMD. These results are in accordance with those of previous studies regarding the association between uric acid level and metabolic syndrome. In a cohort study of 811 otherwise healthy overweight or obese people, an independent significant positive correlation between triglycerides and uric acid levels was found, and the correlation between uric acid and metabolic syndrome was significant [45]. Another study that investigated the risk factors for hyperuricemia in postmenopausal women reported that triglyceride levels and waist circumference correlated with high serum uric acid levels (≥4 mg/dL) [46].

There are several limitations in this study. First, it was a single-center study involving participants who underwent regular check-ups at a gynecological endocrinology menopause clinic; therefore, a selection bias may have existed. For this reason, we introduced strict inclusion and exclusion criteria. Second, the duration of menopause, which averaged 8.9 years in this study, was not subdivided for analysis, and it may have affected the serum uric acid levels and BMD. Third, 328 menopausal women for cross-sectional analysis, including 186 women for longitudinal analysis with follow-up BMD examinations, were enrolled, which is a smaller number compared to populations enrolled in previous studies. Fourth, there were also limitations and risk of bias which could be attributed to the retrospective cross-sectional design with limited numbers of participants for longitudinal analysis because of skewed data, and we could not clarify all the interferences of multiple metabolic indicators with BMD. We did not adjust for individual dietary intake of purine-containing foods, alcohol consumption, lifestyle, exercising habits, and smoking status which affect the serum uric acid level and BMD. Finally, we cannot generalize our results and findings to different populations because there are evolving issues on racial and ethnic differences regarding uric acid action. 

Nevertheless, to the best of our knowledge, this study was the first longitudinal study in Asian postmenopausal women on the association between serum uric acid levels and BMD change and also the first report to assess the association of bisphosphonate use and serum uric acid levels in healthy postmenopausal women. There was no significant difference in serum uric acid levels between bisphosphonate users and non-users. In comparison with HRT users, the difference in serum uric acid levels of bisphosphonate users who concurrently used EPT was also not significant. However, further prospective studies with large populations are needed to confirm this study’s findings regarding the correlation between bisphosphonate use and serum uric acid levels in postmenopausal women who use HRT because of the small size of this study’s population.

In conclusion, this study suggests that there is no association between serum uric acid levels and BMD in postmenopausal women. Identifying the effect and mechanism of action of bisphosphonates on serum uric acid in postmenopausal women who use HRT would be a challenge for future researchers, and this study could serve as a basis.

## Figures and Tables

**Table 1:** Baseline characteristics of the patients.

	Total (n = 328)	Uric Acid			*p* ^b^
		Tertile 1 ^a^ (n = 113)	Tertile 2 ^a^ (n = 102)	Tertile 3 ^a^ (n = 113)	
Uric acid					
Mean ± SD	4.6 ± 1.0	3.5 ± 0.5	4.5 ± 0.2	5.6 ± 0.7	<0.001
Median (range)	4.5 (2.2–8.0)	3.7 (2.2–4.1)	4.5 (4.2–4.8)	5.4 (4.9–8.0)	
Age (years)					
Mean ± SD	57.3 ± 6.5	57.8 ± 6.6	56.3 ± 6.0	57.7 ± 6.8	0.09
Median (range)	57.0 (36.0–81.0)	57.0 (36.0–75.0)	55.0 (40.0–71.0)	57.0 (41.0–81.0)	
Body mass index (kg/m^2^) (n = 270)					
Mean ± SD	23.1 ± 2.8	22.4 ± 2.8	23.1 ± 3.0	23.7 ± 2.4	0.00
Median (range)	22.9 (15.3–34.2)	22.1 (15.3–31.1)	23.0 (17.2–34.2)	23.8 (17.8–32.5)	
Body fat mass (n = 270)					
Mean ± SD	32.7 ± 6.2	31.9 ± 6.5	33.4 ± 6.1	33.0 ± 5.8	0.28
Median (range)	33.5 (3.2–60.7)	31.4 (12.5–46.4)	33.9 (20.1–60.7)	34.0 (3.2–43.1)	
Abdominal obesity (n = 270)					
Mean ± SD	0.88 ± 0.05	0.88 ± 0.05	0.88 ± 0.05	0.89 ± 0.04	0.06
Median (range)	0.89 (0.73–1.01)	0.89 (0.74–1.01)	0.88 (0.73–1.01)	0.89 (0.79–0.98)	
Total lean body mass (n = 270)					
Mean ± SD	37.8 ± 4.2	37.1 ± 4.0	37.8 ± 4.2	38.4 ± 4.2	0.15
Median (range)	37.4 (21.1–51.3)	36.7 (29.0–51.3)	37.7 (21.1–47.5)	38.1 (31.4–51.0)	
Drug use (%)					
HRT					
No	241 (73.5)	84 (74.3)	77 (75.5)	80 (70.8)	0.72
Yes	87 (26.5)	29 (25.7)	25 (24.5)	33 (29.2)	
Bisphosphonates					
No	271 (82.6)	91 (80.5)	86 (84.3)	94 (83.19)	0.75
Yes	57 (17.4)	22 (19.5)	16 (15.7)	19 (16.81)	
Hyperlipidemia					
No	244 (74.4)	85 (75.2)	75 (73.5)	84 (74.34)	0.96
Yes	84 (25.6)	28 (24.8)	27 (26.5)	29 (25.66)	
Fasting blood sugar (mg/dL) (n = 295)					
Mean ± SD	99.4 ± 11.9	99.4 ± 13.4	99.2 ± 11.6	99.7 ± 10.7	0.69
Median (range)	97.0 (79.0–163.0)	97.0 (79.0–162.0)	97.0 (79.0–163.0)	98.0 (83.0–143.0)	
Blood urea nitrogen (mg/dL) (n = 289)					
Mean ± SD	14.8 ± 3.6	14.4 ± 3.8	14.3 ± 3.2	15.7 ± 3.7	0.01
Median (range)	14.3 (6.6–26.7)	13.9 (7.0–26.7)	13.9 (7.5–21.5)	15.3 (6.6–25.7)	
Creatinine (mg/dL) (n = 295)					
Mean ± SD	0.7 ± 0.3	0.6 ± 0.1	0.7 ± 0.1	0.7 ± 0.5	0.00
Median (range)	0.7 (0.4–6.1)	0.6 (0.4–1.1)	0.7 (0.4–0.9)	0.7 (0.5–6.1)	
Alkaline phosphatase (U/L) (n = 261)					
Mean ± SD	177.9 ± 52.9	178.4 ± 64.0	178.7 ± 45.4	176.5 ± 46.9	0.91
Median (range)	170.0 (40.0–475.0)	169.0 (40.0–475.0)	167.5 (94.0–309.0)	175.0 (87.0–334.0)	
Total cholesterol (mg/dL) (n = 293)					
Mean ± SD	200.7 ± 40.3	197.2 ± 39.0	206.0 ± 40.4	199.1 ± 41.3	0.34
Median (range)	199.0 (118.0–336.0)	197.0 (118.0–335.0)	200.0 (129.0–336.0)	196.0 (121.0–295.0)	
Triglycerides (mg/dL) (n = 288)					
Mean ± SD	113.0 ± 60.3	96.5 ± 45.4	111.3 ± 58.4	129.8 ± 69.5	0.00
Median (range)	99.0 (40.0–408.0)	83.0 (40.0–259.0)	96.0 (42.0–376.0)	109.0 (40.0–408.0)	
LDL cholesterol (mg/dL) (n = 277)					
Mean ± SD	115.1 ± 35.2	112.2 ± 33.1	118.5 ± 35.5	114.8 ± 36.9	0.47
Median (range)	114.0 (32.0–233.0)	112.5 (32.0–233.0)	115.5 (39.0–220.0)	111.0 (52.0–201.0)	
Insulin (n = 253)					
Mean ± SD	5.7 ± 3.3	5.3 ± 3.0	6.0 ± 3.7	5.9 ± 3.0	0.25
Median (range)	5.1 (0.5–26.8)	4.6 (0.5–18.2)	5.2 (1.2–26.8)	5.4 (1.7–18.4)	
PYR-D (n = 289)					
Mean ± SD	11.6 ± 21.9	13.0 ± 26.8	10.6 ± 21.2	11.1 ± 16.9	0.38
Median (range)	6.8 (2.3–241.0)	6.9 (3.0–241.0)	6.9 (2.3–184.5)	6.5 (2.6–114.4)	
Osteocalcin (n = 289)					
Mean ± SD	18.3 ± 7.4	17.8 ± 7.7	19.4 ± 7.7	17.7 ± 6.6	0.23
Median (range)	16.6 (5.8–46.8)	15.8 (5.8–46.8)	18.1 (6.3–39.3)	17.1 (6.3–33.8)	
25-OH vitamin D (n = 189)					
Mean ± SD	21.6 ± 10.7	22.6 ± 11.7	20.4 ± 9.7	21.8 ± 10.8	0.62
Median (range)	18.6 (6.9–64.8)	20.3 (6.9–58.3)	18.1 (7.6–53.6)	18.6 (9.0–64.8)	
Follicle-stimulating hormone (n = 133)					
Mean ± SD	76.0 ± 30.8	76.1 ± 31.2	78.3 ± 31.5	73.5 ± 30.3	0.74
Median (range)	72.8 (10.1–159.9)	70.9 (12.0–137.6)	72.8 (10.1–159.9)	72.8 (18.7–153.9)	
Estradiol (n = 267)					
Mean ± SD	22.7 ± 32.4	21.7 ± 26.3	24.0 ± 40.7	22.6 ± 29.1	0.69
Median (range)	12.0 (0.0–248.0)	12.0 (0.0–119.0)	10.0 (0.0–248.0)	12.0 (0.0–197.0)	
Thyroid function test					
T3 (n = 140)					
Mean ± SD	1.0 ± 0.6	1.0 ± 0.2	1.1 ± 1.0	1.0 ± 0.2	0.50
Median (range)	1.0 (0.7–8.0)	1.0 (0.7–1.4)	1.0 (0.7–8.0)	1.0 (0.7–1.5)	
T4 (n = 138)					
Mean ± SD	0.9 ± 0.2	0.9 ± 0.2	0.9 ± 0.2	0.9 ± 0.2	0.80
Median (range)	0.9 (0.5–2.0)	0.8 (0.6–1.7)	0.9 (0.5–1.7)	0.8 (0.6–2.0)	
TSH (n = 139)					
Mean ± SD	2.7 ± 3.0	2.9 ± 4.1	2.6 ± 2.3	2.5 ± 2.6	0.98
Median (range)	2.0 (0.3–26.7)	2.0 (0.6–26.7)	1.9 (0.3–13.5)	2.0 (0.7–17.0)	

Abbreviations: SD, standard deviation; LDL, low-density lipoprotein; PYR-D, pyridinoline; T3, triiodothyronine; T4, free thyroxine; TSH, thyroid-stimulating hormone. ^a^ Tertile 1: ≤4.1 mg/dL; tertile 2: >4.1 and ≤4.8 mg/dL; tertile 3: >4.8 mg/dL; ^b^ *p*-values were calculated using the Kruskal–Wallis test or chi-squared tests. The data are presented as n (%) for categorical variables, unless otherwise indicated.

**Table 2:** Distribution of BMD according to the serum uric acid level at baseline (n = 328) and follow-up (n = 185).

	**Total** **(n = 328)**	**Uric Acid**			***p* ^b^**
		**Tertile 1 ^a^** **(n = 113)**	**Tertile 2 ^a^** **(n = 102)**	**Tertile 3 ^a^** **(n = 113)**	
BMD					
Spine					
Mean ± SD	1.065 ± 0.144	1.050 ± 0.131	1.060 ± 0.160	1.084 ± 0.140	0.22
Median (range)	1.044 (0.709–1.668)	1.024 (0.756–1.477)	1.042 (0.709–1.668)	1.066 (0.843–1.486)	
Femoral neck					
Mean ± SD	0.850 ± 0.108	0.837 ± 0.110	0.849 ± 0.096	0.863 ± 0.115	0.28
Median (range)	0.847 (0.251–1.247)	0.843 (0.251–1.092)	0.844 (0.625–1.053)	0.856 (0.585–1.247)	
	**Total** **(n = 185)**	**Uric Acid**			***p***
		**Tertile 1** **(n = 61)**	**Tertile 2** **(n = 64)**	**Tertile 3** **(n = 60)**	
At baseline					
BMD					
Spine (n = 185)					
Mean ± SD	1.059 ± 0.142	1.034 ± 0.121	1.063 ± 0.152	1.079 ± 0.148	0.32
Median (range)	1.042 (0.712–1.563)	1.017 (0.764–1.307)	1.042 (0.712–1.563)	1.063 (0.843–1.486)	
Femoral neck (n = 184)					
Mean ± SD	0.844 ± 0.102	0.833 ± 0.091	0.848 ± 0.098	0.850 ± 0.117	0.51
Median (range)	0.837 (0.580–1.247)	0.827 (0.580–1.026)	0.841 (0.627–1.071)	0.836 (0.585–1.247)	
Visit 1					
BMD					
Spine (n = 185)					
Mean ± SD	1.058 ± 0.137	1.030 ± 0.117	1.062 ± 0.152	1.083 ± 0.136	0.14
Median (range)	1.048 (0.709–1.540)	1.031 (0.803–1.350)	1.045 (0.709–1.540)	1.058 (0.879–1.451)	
Femoral neck (n = 184)					
Mean ± SD	0.831 ± 0.115	0.827 ± 0.085	0.834 ± 0.108	0.832 ± 0.147	0.65
Median (range)	0.836 (0.027–1.081)	0.816 (0.675–1.011)	0.844 (0.570–1.081)	0.843 (0.027–1.050)	
Median difference in change in BMD (Δbaseline–visit 1)					
ΔBMD					
Spine (n = 185)					
Mean ± SD	0.001 ± 0.056	0.004 ± 0.066	0.001 ± 0.050	−0.004 ± 0.052	0.95
Median (range)	−0.001 (−0.182–0.425)	−0.002 (−0.116–0.425)	0.001 (−0.103–0.142)	−0.004 (–0.182–0.086)	
Femoral neck (n = 183)					
Mean ± SD	0.013 ± 0.087	0.006 ± 0.049	0.014 ± 0.058	0.019 ± 0.132	0.34
Median (range)	0.005 (−0.188–0.978)	0.008 (−0.188–0.254)	0.005 (−0.135–0.217)	−0.003 (−0.055–0.978)	

Abbreviations: BMD, bone mineral density; SD, standard deviation. ^a^ Tertile 1: ≤4.2 mg/dL; tertile 2: >4.2 and ≤5 mg/dL; tertile 3: >5 mg/dL; ^b^ *p*-values are calculated using the Kruskal–Wallis test with the Dwass–Steel–Critchlow–Fligner (DSCF) post hoc test.

**Table 3:** Simple, multiple linear regression.

	log-BMD, Spine				log-BMD, Femoral Neck			
	β ± SE	*p*	Adjusted β ± SE	*p*	β ± SE	*p*	Adjusted β ± SE	*p*
log-Uric acid	0.04 ± 0.03	0.270	0.01 ± 0.03	0.706	−0.0004 ± 0.03	0.990	−0.04 ± 0.04	0.327
Age			−0.003 ± 0.001	0.027			−0.003 ± 0.002	0.032
BMI			0.02 ± 0.01	0.003			0.01 ± 0.01	0.305
log-Body fat mass			−0.01 ± 0.04	0.885			−0.01 ± 0.05	0.894
log-Abdominal fatness			−0.72 ± 0.26	0.005			−0.08 ± 0.29	0.792
log-Total lean body mass			0.12 ± 0.09	0.177			0.19 ± 0.10	0.051
R^2^	0.004		0.141		0.000		0.081	

Data of uric acid, body fat mass, abdominal fatness, total lean body mass, and BMD showed skewed distribution and therefore were log-transformed before analysis.

**Table 4:** Association of HRT and bisphosphonates with uric acid level.

	Uric Acid Level		
	Mean ± SD	Median (Range)	*p* ^a^
Type of treatment HRT			
No (n = 241)	4.5 ± 1.0	4.5 (2.2–8.0)	0.38
Yes (n = 87)	4.6 ± 1.1	4.5 (2.3–7.8)	
Bisphosphonates			
No (n = 271)	4.6 ± 1.0	4.5 (2.2–8.0)	>0.99
Yes (n = 57)	4.6 ± 1.0	4.5 (2.8–7.0)	
Hyperlipidemia			
No (n = 244)	4.5 ± 1.0	4.5 (2.2–8.0)	0.57
Yes (n = 84)	4.6 ± 1.0	4.5 (2.4–7.8)	
HRT subtypes			
E only (n = 28)	4.9 ± 1.0	4.7 (3.2–7.8)	0.28
EPT (n = 30)	4.4 ± 1.1	4.4 (2.3–7.0)	
EPT + bisphosphonates (n = 13)	4.6 ± 1.2	4.3 (2.7–7.0)	
Tibolone (n = 16)	4.7 ± 1.0	5.1 (2.5–6.0)	
HRT non-users (n = 241)	4.5 ± 1.0	4.5 (2.2–8.0)	

Abbreviations: HRT, hormone replacement therapy; E, estrogen; EPT, estrogen and progesterone treatment; SD, standard deviation. The data are presented as n (%) for categorical variables, unless otherwise indicated. ^a^ To determine *p*-values, the Wilcoxon rank sum test or the Kruskal–Wallis test with Dwass–Steel–Critchlow–Fligner (DSCF) post hoc test were used. Additional *p*-values: E only vs. EPT: 0.276; E only vs. EPT + bisphosphonates: 0.772; E only vs. tibolone: >0.999; E only vs. HRT non-users: 0.370; EPT vs. EPT + bisphosphonates: >0.999; EPT vs. tibolone: 0.751; EPT vs. HRT non-users: 0.940; EPT + bisphosphonates vs. tibolone: 0.979; EPT + bisphosphonates vs. HRT non-users: >0.999; tibolone vs. hormone replacement therapy non-users: 0.844.

## Data Availability

The data presented in this study are available in the Supplementary Materials.

## Supplementary Materials

The following are available online at https://www.mdpi.com/article/10.3390/healthcare9121681/s1, Table S1: The data presented in this study.
